# Genomic Variation: Lessons Learned from Whole-Genome CNV Analysis

**DOI:** 10.1007/s40142-014-0048-4

**Published:** 2014-07-18

**Authors:** Erin Rooney Riggs, David H. Ledbetter, Christa Lese Martin

**Affiliations:** 1 Autism and Developmental Medicine Institute, Geisinger Health System, 120 Hamm Drive, Suite 2A, Lewisburg, PA 17837 USA; 2 Geisinger Health System, 100 N Academy Ave., Danville, PA 17822 USA

**Keywords:** Copy number variants, Structural variation, Gene dosage, Chromosomal microarray

## Abstract

One of the most fundamental goals of the study of human genetics was to determine the relationship between genomic variation and human disease. The effects of large-scale structural variation, such as aneuploidy and other cytogenetically visible imbalances, as well as sequence-level variation, have been studied for several decades. However, compared to these, the impact of submicroscopic copy number variants (CNV) has only recently been appreciated. Despite this, lessons learned from the study of CNVs have already proven significant and broadly applicable. From expanding the concept of normal human variation to providing concrete examples of the utility of genomics in clinical care and challenging notions of the genetic architecture of complex disease, CNVs have provided valuable insights into the genomics of human health and development.

## Introduction

The physical map and DNA sequence derived from the Human Genome Project [1] revolutionized cytogenetic testing by providing the ability to detect submicroscopic imbalances across the genome. Technologic advances, namely the advent of comparative genomic hybridization, allowed for the detection of CNVs in the research setting in the 1990s [2–5]; subsequent improvements in the test design gave chromosomal microarray (CMA) more utility in the clinical diagnostic setting. These advances included the addition of large genomic clones (e.g., bacterial artificial chromosomes, or BACs) to allow for the detection of single-copy losses or gains [6] followed by the replacement of genomic clones with synthetic oligonucleotides [7] for genome-wide interrogation and more precise identification of breakpoints. By the late 2000s, oligonucleotide CMA designs were implemented for clinical testing that included both targeted (representing known clinically relevant regions) and genome-wide backbone coverage [8]. This design schema enabled CMA to identify all imbalances detectable by karyotype plus submicroscopic CNVs, thus surpassing the diagnostic yield of a G-banded karyotype [9••].

At the time CMA was first being implemented in the clinic, the role of CNVs in human disease was still largely unclear—the mechanisms of formation were not completely understood, and the clinical significance of many novel findings was frustratingly uncertain. Over time, through intense examination and data sharing, the roles, mechanisms, and significance of CNVs have become clearer, laying the foundation for discovery in the next-generation sequencing era.

### Expanding the Scope of Normal Human Variation

Although structural variation in normal individuals has been appreciated for decades at the microscopic level [10], the extent to which the human genome was subjected to submicroscopic copy number variation was not realized until 2004. Studies using CMA technology revealed CNVs throughout the genomes of normal individuals, several of which were present in >10 % of the individuals studied [11, 12]. Indeed, CNVs are thought to account for ~1 % of the variation between two individuals; in contrast, single nucleotide polymorphisms (SNPs) are thought to account for approximately 0.1 % [13].

Studies documenting common areas of normal structural variation continue to serve as valuable resources for those evaluating the clinical significance of CNVs; it has historically been assumed that CNVs identified in “normal” populations could be classified as “likely benign” or “benign.” Early guidelines for the clinical interpretation of CNVs proposed that CNVs inherited from reportedly normal parents could “probably” be considered benign [9••]. However, assumptions such as these should be made with caution and in the context of the family’s clinical presentation. There are many examples of “pathogenic” CNVs exhibiting reduced penetrance and/or variable expressivity, and many instances in which a more severely affected child has inherited a CNV from a seemingly normal parent. The 22q11.2 deletion (del) syndrome is a classic example of this phenomenon [14], though few would argue about the pathogenicity of this particular CNV.

A more fitting example might be del 15q11.2, including the region between breakpoints (BP) 1–2. A number of case–control studies have demonstrated that this deletion is enriched in cases as compared to controls [15•–17]. Nonetheless, the fact that it has been observed in control individuals and unaffected relatives, coupled with the broad spectrum of associated phenotypes (developmental delay [15•, 16], schizophrenia [18], epilepsy [17, 19], etc.), has anecdotally resulted in some hesitances to classify it as “pathogenic.” However, a recent study of control individuals found to carry CNVs previously associated with neuropsychiatric disorders, such as autism spectrum disorders (ASD) and schizophrenia (including del 15q11.2 BP 1–2), showed that these individuals performed at a level between that of schizophrenic patients and population controls on a series of cognitive measures, even though they had never received a formal neuropsychiatric diagnosis [20••]. This observation gives more credence to the interpretation of del 15q11.2 as pathogenic with variable expressivity but, more importantly, puts forth the idea that CNVs observed in seemingly normal populations could indeed be conferring varying levels of clinical effects, challenging the notion that variation found in normal populations is predominantly benign. Therefore, quantitative measures assessing neurodevelopmental phenotypes, such as cognition and behavior, may be more helpful than broad categorical diagnoses (e.g., affected versus unaffected) when trying to establish the effect of genomic variation [21].

### A Paradigm Shift in Clinical Genetic Care

While certain CNVs are common within the “normal” population, others have been associated with disorders of human health and development. Several genomic disorders were identified with the advent of high-resolution chromosome banding and fluorescence in situ hybridization (FISH) technologies (Prader Willi/Angelman syndromes [OMIM 176270/105830]; Miller-Dieker syndrome [OMIM 247200], Williams-Beuren syndrome [OMIM 194050], etc.); however, the widespread use of CMA allowed for the identification of numerous others at a remarkable pace [22–26], often before a clear phenotypic picture had emerged.

As CMA design evolved to include targeted coverage of clinically relevant regions and uniformly spaced backbone coverage throughout the euchromatic regions of each chromosome [8], CMA truly became a “genome-wide” assay. Although the G-banded karyotype was essentially the first genome-wide assay in the most basic sense of the term, CMA provided clinicians with a way to interrogate the entire genome with a single, high-resolution assay. Before this, genetic diagnoses were made based upon a clinician’s observations of the patients presenting phenotype, and that phenotype’s consistency (or lack thereof) with previously described genetic syndromes. Even with the advent of sequence-based genetic testing, diagnoses still relied on the clinician’s ability to deduce a plausible set of differential diagnoses from the observed phenotype and select the correct gene(s) to test, if clinically available. Diagnostic testing under this paradigm required an a priori idea of the underlying diagnosis and causative mechanism, and testing each of the possible differential diagnoses was undertaken separately. This approach was ineffective if the suspected clinical diagnosis was incorrect or if the causative mechanism was unknown, leaving many patients without a confirmed genetic diagnosis. It naturally followed that those presenting with classical symptoms of well-described genetic conditions had the best chance of obtaining a diagnosis, while those with more ambiguous symptoms or with rarely or never described conditions remained undiagnosed or were misdiagnosed.

With CMA, no priori predictions of the patient’s diagnosis are required, making the test particularly appealing for use in those with non-specific symptoms, including developmental delays, ASD, and congenital anomalies. By evaluating the entire genome at once, both previously described syndromes and novel etiologies could be identified. CMA becoming recognized as a first-tier test for these groups of individuals [27•] represented a paradigm shift in the diagnosis of genetic disorders from “phenotype-first,” where clinicians used the patient’s phenotype to guide decisions about which genetic tests to order, to “genotype-first,” where clinicians used the patient’s genotype, to guide their evaluation and management.

CMA results, expressed in specific genomic coordinates, also afforded clinicians with the ability to truly integrate information garnered from personal genotype into medical care. With more precise breakpoints for patients’ CNVs, laboratories and clinicians can determine which genes are involved in a CNV. Using available information about the gene(s) phenotypic effects and dosage sensitivity, one can extrapolate which of the patient’s presenting features could be explained by the CNV, as well as health issues that the patient could be at risk for in the future. Such health issues may be associated with specific management recommendations which could be implemented for a patient before they were even symptomatic.

For example, an individual could present to medical attention for developmental delay, and CMA results demonstrate a large deletion on chromosome 7q involving the *KCNH2* gene associated with long-QT syndrome 2 (OMIM 613688) [28]. Such results would indicate that the individual is at high risk for developing this disorder, which is associated with cardiac arrhythmia and sudden death. A referral to a cardiologist is then warranted for evaluation and management of this risk, something that was likely not expected in the context of the original presenting symptoms (i.e., an incidental finding). Examples such as this one are not infrequent, particularly given the number of potentially actionable genes with dosage sensitivity, such as cancer predisposition genes, which are covered as part of standard array designs. One study using data from the International Standards for Cytogenomic Arrays (ISCA) consortium database estimated that ~7 % of reported cases involved a region of the genome associated with some types of published medical management recommendations, demonstrating the clinical actionability of CMA test results [29].

### Key Contributor to Complex Conditions

Additionally, CNVs have been identified as important contributors to complex conditions, such as ASD. Heritability estimates have varied for ASD, but have been reported as high as 90 % [30]. Identifying the genetic basis of these types of conditions through traditional methods (such as linkage analysis) has been challenging due to their extensive genetic and phenotypic heterogeneity. Linkage studies identified several SNPs associated with ASD, but all with relatively low effect size [30]. Likewise, genome-wide association studies (GWAS) undertaken to identify common risk variants have been largely unsuccessful. Although several common risk variants have been identified, the results have been difficult to replicate and were associated with odds ratios less than 1.23 [1, 32], making it clear that these methods were missing variants of moderate–high effect.

As these populations began to be evaluated for CNVs, the significant contribution of structural variation to ASD became apparent. Many CNVs have been identified among individuals with ASD [33–35], and some recurrent CNVs (mediated by segmental duplications) have even reached statistical significance in large-scale case–control studies [34]. Although none of these CNVs individually account for more than 1 % of ASD cases, as a group, structural variants play a significant role in the development of this disorder, providing a genetic diagnosis in 5–10 % of cases. CNVs have been reported to confer more than 3 times the level of risk attributed to SNPs identified through GWAS [32, 36].

Similar observations have been made in other complex disorders, such as intellectual disability, schizophrenia, epilepsy, and cardiac defects (presented in this issue). Interestingly, many of the same CNVs are being identified among phenotypes which were previously considered distinct. These observations have led some to consider whether these phenotypes may actually represent aspects of an etiologically related continuum, such as the developmental brain dysfunction model described for neurodevelopmental disorders [21].

## Conclusion

Identifying an underlying genetic etiology for any human phenotype is invaluable, both to the individual patient and to the research community as a whole. For the individual patient, receiving a specific genetic diagnosis can end the taxing diagnostic odyssey, contribute to current and future medical management, and impact family planning considerations. For the research community, molecularly defining previously uncharacterized disorders provides the opportunity to learn more about gene function, gene–gene interactions, and genotype–phenotype correlations, which will ultimately lead to targeted therapeutics.

The lessons learned from CMA and copy number variation have contributed greatly to both the ability to diagnose individuals and to the knowledge base surrounding the mechanisms of human disease. As whole-exome and whole-genome technologies become more accessible, the cycle of discovery and knowledge assimilation will continue to accelerate; new variants will be discovered, new mechanisms will be deduced, and our perception of clinical genomics will evolve. These types of genome-wide assays have already and will continue to identify genomic variants with immediately appreciable effects on human health and development. However, they will also continue to identify variants of uncertain clinical significance, as our ability to identify variation is currently beyond our ability to accurately interpret its possible phenotypic consequences.

Various databases designed to catalog and make publicly available structural variation data from both normal and affected populations (Database of Genomic Variation [37] for normal populations; the ISCA Consortium [9••], DECIPHER [38], ECARUCA [39], etc. for affected populations) have been established. These databases continue to serve as valuable resources for laboratories and clinicians as they interpret CNVs observed in patients on a daily basis. As technologic advances move toward the ability to detect both structural- and sequence-level data from the same testing platform, similar resources are needed to make information about both types of variation readily accessible to both the research and clinical communities. ClinVar is a resource housed within the National Center for Biotechnology Information (NCBI) that collects information about the relationships between human variation (both structural and sequence level) and human disease. Large-scale efforts are underway through the International Collaboration for Clinical Genomics (ICCG) [40] to facilitate the submission of clinical laboratory data from CMA- and sequencing-based tests into this database, with plans to ultimately include whole-exome and whole-genome data. These efforts are part of a larger collaboration, the Clinical Genomics Resource (ClinGen), which aims to use this information, coupled with manual and machine learning-based curation efforts, to create a “clinical genome,” or catalog of variants known to be relevant to clinical care.

The scope of knowledge regarding the nature of structural variation and its relationship to human health has expanded dramatically over the last several decades; with increased usage of whole-genome and whole-exome sequencing, it is expected that the same will occur for sequence-level variation. Increased data sharing and collaboration will lead to substantial progress in understanding the relationships between variants and disease, making personalized genomic medicine a reality.
